# Hypertension and breast cancer risk: a systematic review and meta-analysis

**DOI:** 10.1038/srep44877

**Published:** 2017-03-20

**Authors:** Hedong Han, Wei Guo, Wentao Shi, Yamei Yu, Yunshuo Zhang, Xiaofei Ye, Jia He

**Affiliations:** 1 Department of Health Statistics, Second Military Medical University, Shanghai 200433, China; 2 Department of Cardiology, Changhai Hospital, Second Military Medical University, Shanghai 200433, China; 3 Department of Pathology, Changhai Hospital, Second Military Medical University, Shanghai 200433, China

## Abstract

Observational studies examining the relationship between hypertension and breast cancer risk have reported conflicting findings. We conducted this systematic review and meta-analysis to summarize the evidence regarding the association between hypertension and risk of breast cancer. Eligible studies were identified through a comprehensive literature search of PubMed, EMBASE, and the Cochrane library until August 2016. We included observational studies that reported relative risks (RR) with corresponding 95% confidence intervals (CIs). Results from individual studies were pooled by using a random-effects model. 29 articles of 30 studies, with totally 11643 cases of breast cancer, were eligible for inclusion in the meta-analysis. We observed a statistically significant association between hypertension and increased breast cancer risk (RR: 1.15; 95% CI: 1.08, 1.22). In the subgroup analysis, we found a positive association between hypertension and breast cancer incidence among postmenopausal women (RR: 1.20; 95% CI: 1.09, 1.31). In contrast, hypertension was not associated with risk of breast cancer among premenopausal women (RR: 0.97; 95% CI: 0.84, 1.12) and Asian population (RR: 1.07; 95% CI: 0.94, 1.22).This meta-analysis collectively suggests a significantly association between hypertension and breast cancer risk, specifically for postmenopausal hypertensive women.

Breast cancer is the second most common cancer overall (1.7 million cases) and ranks 5th as cause of death (522,000 cases) in 2012 worldwide^1^. Both incidence and mortality from breast cancer in women vary among populations around the world, with higher rates in most developed countries than in less developed countries^2^. The incidence rate of breast cancer has also been increasing rapidly in Asian countries^3^, particularly a steady growth rate of 3–5% annually has been reported in China for the past three decades^4^. Studies have suggested that several factors including age, starting menstruating early or having a late menopause, family history and genetic factors, previous benign breast disease, radiation, obesity, oral contraceptives, hormonal replacement therapy and diabetes mellitus are associated with high breast cancer risk^2^,^5^,^6^. Hypertension, a common chronic disease and major risk factor for cardiac cerebral vascular disease and chronic kidney disease, has also been implicated as a risk factor for breast cancer^7^.

However, case-control and cohort studies that examined the relationship between hypertension and breast cancer risk in women have given inconclusive results. One cohort study, one nested case-control study and ten case-control studies^8^,^9^,^10^,^11^,^12^,^13^,^14^,^15^,^16^,^17^,^18^ suggested that hypertension was related to increased risk of breast cancer, while other studies^19^,^20^,^21^,^22^,^23^,^24^,^25^,^26^,^27^,^28^,^29^,^30^,^31^,^32^,^33^,^34^,^35^,^36^ failed to reveal a significant correlation between hypertension and breast cancer risk. A possible reason for this inconsistency could be that individual studies did not provide sufficient power to indicate any benefit or harm. Another explanation was that adjustments varied among the included studies, especially for risk factors such as age, diabetes or obesity. Given the two major concerns of public health and conflicting results discussed above, we conducted a meta-analysis to summarize all available evidence from case-control and cohort studies on the relationship between hypertension and the risk of breast cancer. In the present study, we also examined whether the association between hypertension and the risk of breast cancer differed according to various study characteristics and menopausal status.

## Methods

### Data sources and search strategy

We followed the standard MOOSE^37^ and PRISMA^38^ criteria when conducting this meta-analysis and reporting the results. A systematic literature search was conducted for articles on hypertension and risk of breast cancer, which were published between 1965 and August 2016, using the databases of PubMed, EMBASE, and the Cochrane library. Searches were performed using Medical Subject Heading terms and the free keywords: (“Breast Neoplasms” OR “Breast Cancer” OR “Breast Tumor” OR “Breast Tumors” OR “Breast Carcinoma” OR “Breast Carcinomas”) AND (“Blood Pressure” OR “Hypertension”) AND (“Cohort” OR “Case-control” OR “Case control”). Furthermore, the reference lists of retrieved articles were manually scrutinized to identify potential relevant studies.

### Selection criteria

Two reviewers (H.H. and W.G.) independently evaluated studies for inclusion, and studies were included in the meta-analysis if they met the following criteria: 1) cohort or case control or nested case-control design; 2) the exposure of interest was hypertension (blood pressure higher than corresponding cut-off values or taking antihypertensive medications), and the outcome of interest was breast cancer risk; 3) the risk estimates, such as relative risks, odds ratios, or hazard ratios that could be transformed into relative risks with 95% confidence intervals (CIs) were reported; 4) potential factors were adjusted for breast cancer risk.

### Data extraction and quality assessment

Two reviewers (H.H., W.G.) independently screened the titles and abstracts of the studies to identify all potential eligible studies using a predefined data extraction form. The following data were extracted: the first author’s last name, year of publication, study location, ethnicity, study design, exposure assessment, the definition of hypertension (according to different cut-off values for diagnosis of hypertension), outcome assessment, matched or adjusted factors and NOS score. Quality assessment was conducted using Newcastle–Ottawa Quality Assessment Scale (NOS)^39^ and studies with an NOS score ≥7 were considered high quality.

### Statistical analysis

We examined the relationship between hypertension and the risk of breast cancer based on the effect estimate RR and its 95% CI in each study. Relative risks (RR) were used as the common measure of association across the included studies. We transformed odds ratios (OR) and hazard ratios (HR) into RR. Because the absolute risk of breast cancer is low, the OR and HR approximate the RR^40^,^41^. Random-effect model was used to combine the estimated effects^42^. Statistical heterogeneity among studies was evaluated by using I^2^ statistics^43^. We conducted sensitivity analysis by removing each individual study at a time from the meta-analysis to evaluate the stability of the pooled results and investigate the potential source of the heterogeneity if the heterogeneity was significant. To explore the heterogeneity, we performed subgroup analysis based on study design, number of breast cancer cases, geographical regions, definition of hypertension, whether data was extracted from metabolic syndrome (Mets) studies and study quality. Meta-regression analysis (interaction test) was conducted to explore heterogeneity between subgroups. Publication bias was assessed with the Egger’s regression test^44^ and funnel plot. If publication bias existed, we evaluated the effect of publication bias by using the trim and fill method^45^.

Stata Version 12.0 software (Stata Corp, College Station, TX) was used for all analyses, and a *P*-value < 0.05 was considered to be statistically significant.

## Results

### Description of the selected studies

**Figure 1:**
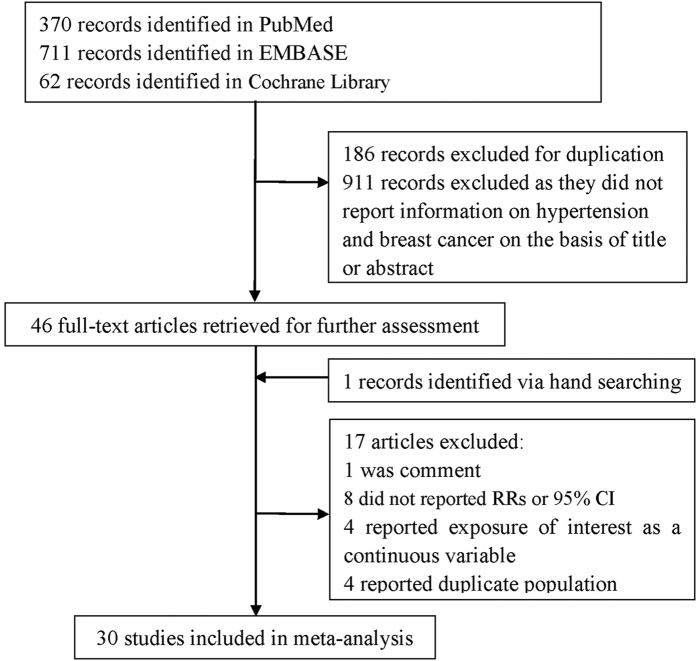
Selection of studies for inclusion in this meta-analysis.

Our literature search identified 1143 articles and 1097 were excluded after review of title or abstract (Fig. 1). Forty-six full-text articles were further reviewed. We excluded seventeen studies due to the following reasons: eight studies did not report RRs or 95% CI; one was comment; four reported exposure of interest as a continuous variable; four reported duplicate population. One study was identified via hand searching and the study was included in our meta-analysis. Thus, the meta-analysis included 30 independent observational studies^8^,^9^,^10^,^11^,^12^,^13^,^14^,^15^,^16^,^17^,^18^,^19^,^20^,^21^,^22^,^23^,^24^,^25^,^26^,^27^,^28^,^29^,^30^,^31^,^32^,^33^,^34^,^35^,^36^ published between 1989 and 2016 with a total of 11643 breast cancer cases. The characteristics of the included studies were summarized in Table 1. Among these studies, eight were conducted in USA, six in Italy, three in China, two in Korea, two in Japan, two in Brazil and seven in other countries. According to the study design, eleven were cohort studies, one was nested case-control studies, and eighteen were case-control studies. Nine studies reported breast cancer patients with premenopausal status and thirteen studies reported breast cancer patients with postmenopausal status. About twenty-two of the included studies provided RRs that were adjusted for age, alcohol consumption, smoking, age at menarche, education, BMI or history of breast cancer. With regard to study quality, the NOS score ranged from 5 to 8.

### Overall analysis of hypertension and breast cancer risk

**Figure 2:**
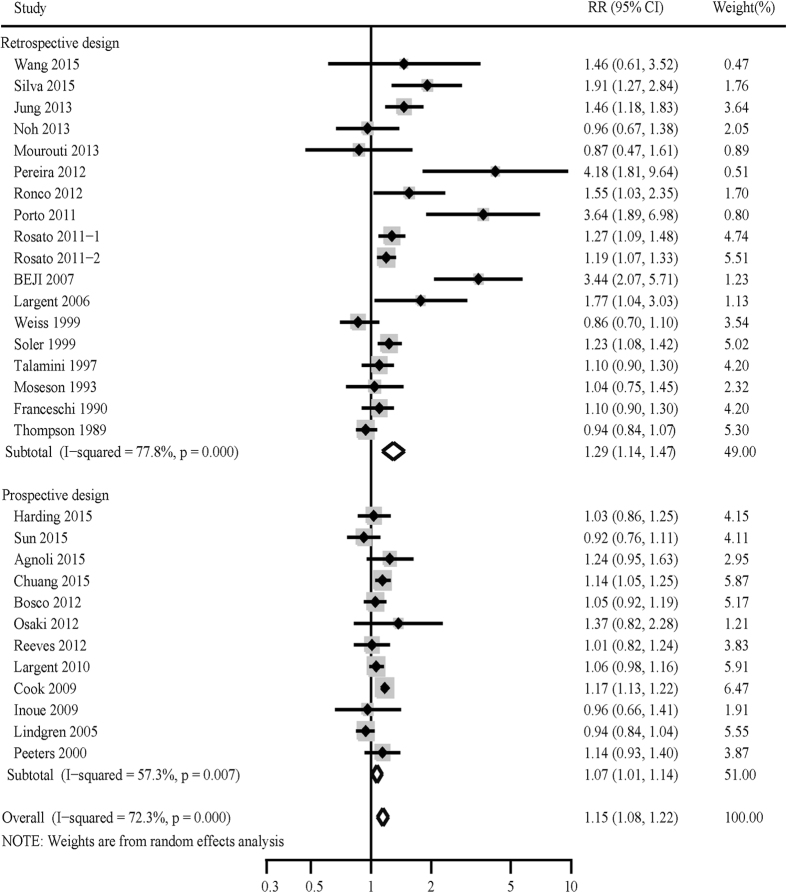
Forest plots of hypertension and the risk of breast cancer.

30 studies regarding the relationship between hypertension and breast cancer risk were included for overall analysis in our meta-analysis. The combined effect estimations (RRs) using random-effects model were presented in Fig. 2. The overall results suggested a statistically significant 15% increase in risk of breast cancer (combined RR: 1.15; 95% CI: 1.08, 1.22). There was statistically significant heterogeneity across the included studies (I^2^ = 72.30%, p < 0.001).

### Subgroup and meta-regression analysis

**Figure 3:**
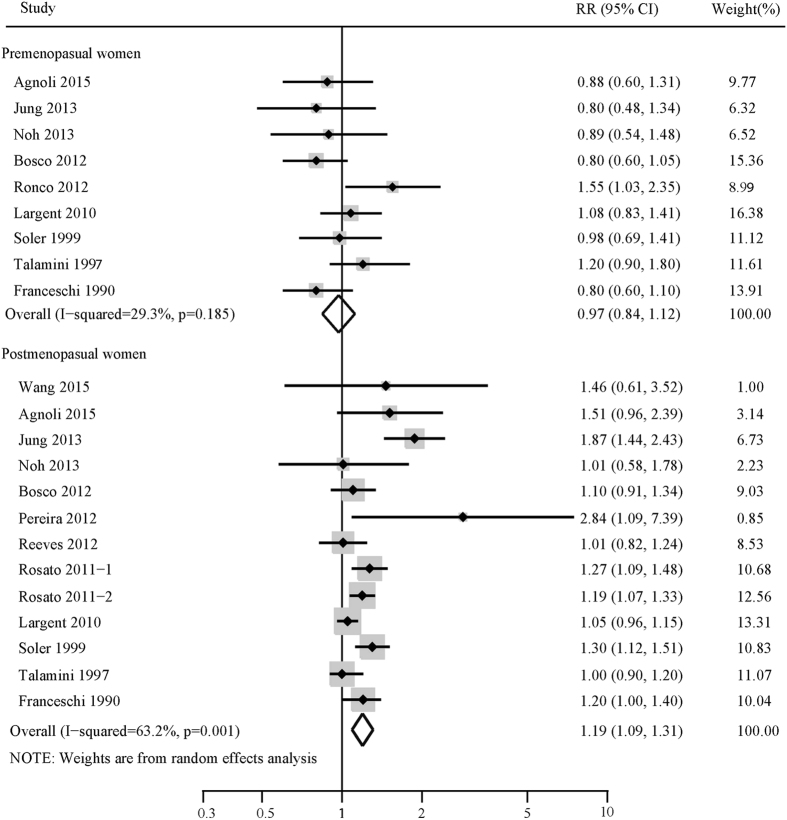
Forest plots of hypertension and the risk of premenopausal and postmenopausal breast cancer.

Subgroup analyses were performed based on study design (prospective, retrospective), definition of hypertension (HP ≥ 160/95mmhg, ≥140/90mmhg or ≥130/85mmhg), menopausal status (premenopausal status, postmenopausal status), geographical region (Asia, America or Europe) and study quality (low quality, high quality). The results of subgroup analysis regarding the relationship between hypertension and risk of breast cancer were shown in Table 2. In the subgroup analysis stratified by study design, statistically significant association was observed in both prospective studies (combined RR: 1.07; 95% CI: 1.01, 1.14, I^2^ = 57.30%, p = 0.01) and retrospective studies (combined RR: 1.29; 95% CI: 1.14, 1.47, I^2^ = 77.80%, p < 0.001). When subgroup analysis was conducted based on geographical region, no significant association was observed in Asian participates (combined RR: 1.07; 95% CI: 0.94, 1.22, I^2^ = 29.40%, p = 0.23). When stratified by definition of hypertension and number of breast cancer cases, no significant association was observed in hypertension defined as BP ≥ 160/90mmhg (combined RR: 1.09; 95% CI: 0.91, 1.31, I^2^ = 79.70%, p = 0.01) and in studies with a smaller number of breast cancer cases (combined RR: 1.20; 95% CI: 0.97, 1.47, I^2^ = 62.90%, p = 0.01). In subgroup analysis concerning menopausal status, the pooled RRs for postmenopausal hypertensive women was 1.20 (95% CI: 1.09, 1.31) with evidence of heterogeneity (I^2^ = 63.20%, p = 0.001), while the pooled RRs for premenopausal hypertensive women was 0.97 (95% CI: 0.84, 1.12) with no evidence of heterogeneity (I^2^ = 29.20%, p = 0.19) (Fig. 3). Meta-regression analysis showed that no variables might account for the heterogeneity across studies (Table 2).

### Publication bias and sensitivity analysis

**Figure 4:**
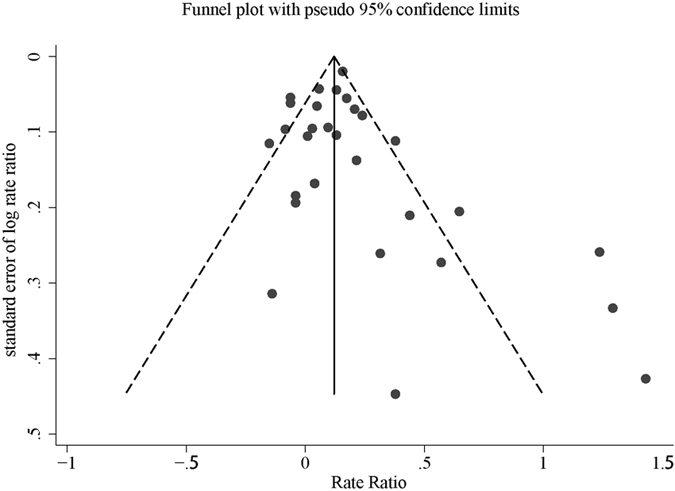
Funnel plots of studies evaluating the risk of breast cancer associated with hypertension.

Publication bias was assessed by funnel plot and Egger’s regression test. Funnel plot shapes demonstrated a symmetrical distribution (Fig. 4) and no evidence of publication bias was detected by the Egger’s regression test (p = 0.26). Sensitivity analysis shown that none of the studies influence the combined results substantially in this meta-analysis.

## Discussion

To our knowledge, this is the first meta-analysis to comprehensively summarize the evidence between hypertension and breast cancer risk. This association has been long reported and assessed, yet with inconsistent results. The meta-analysis demonstrated a positive association between hypertension and breast cancer risk. Summary results showed that women with hypertension may have 15% increased risk of breast cancer. The association between hypertension and breast cancer was consistent for case-control and cohort studies, while the positive association was found in postmenopausal women not in premenopausal women.

The subgroup analyses indicated the effect of increased breast cancer risk was significant only in postmenopausal women, this might be explained by different estrogen metabolism pathways amongst premenopausal and postmenopausal women^46^,^47^. In addition, no significant association detected in smaller sample size studies and studies with a hypertension definition of BP ≥ 160/95mmhg might be due to insufficient statistical power. In our meta-analysis, Asian hypertensive participants did not seem to be associated with increased breast cancer risk, which was corresponding to a relatively low incidence of breast cancer in less developed countries^2^. Subgroup results above were likely the source of heterogeneity in our study, although test for interaction was not statistically significant (*p* = 0.84). As one component of metabolic syndrome, hypertension was reported to associate with increased postmenopausal breast cancer risk in a meta-analysis^48^, with the pooled risk estimations (combined RR:1.13; 95% CI: 1.01, 1.26) that was comparable to our result (combined RR: 1.20; 95% CI: 1.09, 1.31).

Several mechanisms have been proposed for the relationship between hypertension and breast cancer risk. First, breast cancer and hypertension may share common pathophysiological pathway mediated by adipose tissue, which could cause chronic inflammation and further increased the risk of both breast cancer and hypertension^16^,^49^,^50^,^51^. Another possible explanation is that hypertension may increase breast cancer risk by blocking and subsequently modifying apoptosis, thereby affecting the regulation of cell turnover^52^,^53^. In addition, studies reported that this positive association may be confounded by BMI and diabetes^12^,^54^, as significant association appeared to be confined to women with a BMI of at least 25 kg/m^2^ 16. And overweight had been long reported to be associated with elevated estrogen levels and availability and consequently with the risk of postmenopausal breast cancer. However, the analytical model with BMI or diabetes as a covariate indicated that BMI or diabetes did not fully account for the positive association^16^,^17^. Finally, some studies showed women who used antihypertensive medications showed an increased risk of breast cancer compared to those without prescriptions of antihypertensive medications^55^,^56^,^57^. For example, Li *et al*. reported that the use of calcium channel blockers (one of the most commonly prescribed medications for hypertension) was associated with an over two-fold increased risk of breast cancer^57^. However, this positive association was widely doubted^58^,^59^ as the study was based on a quite limited sample size (12 controls, 27 for ductal cases and 31 for lobular cases). In addition, with regard to the design of case-control, potential selection bias, recall bias, and confounding by indication might lead to a biased estimation about the association. Notably, previous meta-analysis and large long-term follow-up cohort studies^60^,^61^,^62^ had confirmed the null association between antihypertensive drugs and breast cancer risk to a large extent. According to the analysis above, antihypertensive medications use was unlikely to alter results in this meta-analysis substantially.

There were several strengths in our study. First, a total of 30 published studies with 11,643 breast cancer cases were pooled in this meta-analysis, which might enhance the statistical power of the data analysis and thus provide more reliable estimates. Second, studies included in this review were not limited to studies with complete cross-table data, but extended to the studies with ORs and 95% CIs. Third, the included studies were conducted in different countries, which made the results more generalizable. Fourth, according to the results of Egger’s test and the funnel plot, we did not find a significant publication bias among included studies. Therefore, we concluded that the results based on the current evidences were relatively convincing.

This meta-analysis also has some limitations. First, we had no access to individual patient-level data, which would have allowed a more reliable assessment of relationship between hypertension and breast cancer risk in different patient groups. Second, hypertension and breast cancer share several risk factors that may confound the relationship. However, confounding cannot be fully excluded because our analyses were based on observational studies. Third, none of the selected studies provided the stages or grades of hypertension and risk of breast cancer; therefore, we were unable to conduct a dose-response analysis to assess the relationship between these variables more precisely. Fourth, the cutoff points for the high and low blood pressure groups were various in the included studies, which might contribute to the heterogeneity and have an influence on the summary risk estimate. Fifth, a high level of heterogeneity was found overall across the analysis. Although we used random-effect models to combine the effect estimations and performed subgroup analysis and meta-regression analysis to explore the sources of heterogeneity, according to our results, these are unlikely to have fully accounted for heterogeneity, suggesting that other potential factors like variation in grades of hypertension, molecular subtypes of breast cancer, exposure assessment or outcome assessment could be involved in this heterogeneity. Finally, breast cancer comprises various histologic subtypes with distinct clinical characteristics, but these were not demonstrated in this study.

In conclusion, our meta-analysis demonstrates that hypertension is associated with increased risk of breast cancer, especially among postmenopausal women. Consequently, health workers should increase the rate of breast cancer screening for postmenopausal hypertensive patients. Meanwhile the, general population is recommended to be involved in behavioral interventions like diet or physical activity to lower the risk of breast cancer by controlling the development of hypertension.

## Additional Information

**How to cite this article:** Han, H. *et al*. Hypertension and breast cancer risk: a systematic review and meta-analysis. *Sci. Rep.* **7**, 44877; doi: 10.1038/srep44877 (2017).

**Publisher's note:** Springer Nature remains neutral with regard to jurisdictional claims in published maps and institutional affiliations.

## Figures and Tables

**Table 1:** Baseline characteristics of all the studies included in the meta-analysis.

Study	Study design	No. of cases	Age (years)	Exposure assessment	Definition of Hypertension	Outcome assessment	Adjustments	NOS
Harding, 2015, Australia	Cohort	549	11−96	Measurement	BP ≥ 130/85mmhg	Cancer registry	Sex, age, smoking, education	7
Wang, 2015, China	Case–control	43	46−61	Measurement	BP ≥ 140/90mmhg	Medical record	Age, education, breastfeeding, family history of breast cancer, age at menarche, age at menopause, number of full-term pregnancies, and age at first birth	6
Sun, 2015, China	Cohort	174	Median: 56.3	Physician-diagnosed	NA	Cancer registry	Age, sex, urbanization level, occupation, income, and comorbidity of diabetes, hyperlipidemia, stroke, ischemic heart disease, chronic obstructive pulmonary disease, alcoholism, alcoholic liver damage, and medication of antihypertensive agents	7
Agnoli, 2015, Italy	Cohort	593	46–61	Measurement	BP ≥ 130/85mmhg	Cancer registry	Menopausal status, parity, age at menarche, smoking, physical activity, education, alcohol	7
Chuang, 2015, China	Nested case-control	1545	Median: 31–40	Database	NA	Medical record	Age, occupation, number of screening test before the index date, the average outpatient visit 6 months prior to the index date	7
Silva, 2015, Brazil	Case-control	984	Mean: 55 for case 50.8 for control	Measurement	BP ≥ 140/90mmhg	Histologically confirmed	Age, sex, smoking	6
Jung, 2013, Korea	Case-control	287	Mean: 48 for case 48.3 for control	Questionnaire	NA	Medical record	Age, age of menarche, pregnancy, age of first pregnancy, and family history of breast cancer	6
Noh, 2013, Korea	Case-control	270	42.2–65.3	Medical record	BP ≥ 130/85mmhg	Routine health examination.	Age, menopausal status, the time of visit to the Health Promotion Center	6
Mourouti, 2013, Greece	Case-control	33	44–68	Questionnaire	BP ≥ 130/85mmhg	Physical biopsy	Work, home places, age	6
Pereira, 2012, Chilean	Case-control	340	Mean: 55.1	Medical record	BP ≥ 140/90mmhg	Histopathologically confirmed	Age, alcohol use, fruit and vegetable intake, physical activity, type 2 diabetes, use of oral contraceptives, use of hormone replacement therapy, obesity, years of formal education, smoking, number of living births	6
Ronco, 2012, Uruguay	Case-control	253	Mean: 40	Measurement	NA	Medical record	Age, education, urban/rural status, age at menarche, number of live births, age at first delivery, years between menarche and first delivery, breastfeeding, oral contraception, family history of breast cancer	7
Bosco, 2012, USA	Cohort	447	Median: 50	Self-reported	BP ≥ 130/85mmhg	Cancer registry	Age, education, BMI at age 18, vigorous activity	8
Reeves, 2012, USA	Cohort	551	≥65	Measurement	BP ≥ 130/85mmhg	Pathology report	Age, current hormone use, family history of breast cancer, and other Mets criteria, BMI	7
Osaki, 2012, Japan	Cohort	77	Mean: 58.6	Medical record	BP ≥ 130/85mmhg	Medical record	Age, smoking status, heavy drinking, presence of metabolic syndrome or pre-metabolic syndrome of each definition	7
Rosato, 2011, Italy	Case–control	1063	33–86	Questionnaires	BP ≥ 130/85mmhg	Medical record	Age, study center, study period, education, alcohol consumption, age at menarche, age at first birth, age at menopause, hormone replacement therapy use, and family history of breast cancer	6
Porto, 2011, Brazil	Case–control	49	40–80	Questionnaires	BP ≥ 130/85mmhg	Medical record	Age	6
Largent, 2010, USA	Cohort	810	Mean: 52.8	Questionnaire	NA	Medical record	Race, family history of breast cancer, age at first full-term pregnancy and number of full-term pregnancies combined variable, hormone therapy and menopausal status combined variable, lifetime physical activity, diabetes, BMI, smoking history, alcohol use, hysterectomy, breastfeeding, and quartiles of percent calories from fat	6
Cook, 2009, USA	Cohort	NA	30–55	Questionnaires	NA	Pathology reports	Parity, age at each birth	6
Inoue, 2009, Japan	Cohort	59	40–69	Measurements	BP ≥ 130/85mmhg	Cancer registry	Age, study area, smoking status, weekly ethanol intake, total serum cholesterol	7
Beji, 2007, Turkey	Case–control	231	28–72	Questionnaire	NA	Histologically confirmed	Age	6
Largent, 2006, USA	Case–control	172	50–75	Questionnaire	NA	Medical record	Age, age at first full-term pregnancy, diabetes, family history of breast or ovarian cancer, smoking, alcohol, BMI, menopausal status and education	7
Lindgren, 2005, Finland	Cohort	307	Mean: 51	Measurements	BP ≥ 160/95mmhg	Cancer registry	Age, year of registration, DBP and SBP as continuous variables, smoking, BMI, use of antihypertensive drugs, functional diagnosis of hypertension and number of children for women	7
Peeters, 2000, Netherlands	Cohort	523	Mean: 57	Measurements	BP ≥ 160/95mmhg	Cancer registry	Age at baseline, height, BMI, smoking, parity, familial breast cancer, use of oral contraceptives	8
Weiss, 1999, USA	Case–control	274	<45	Questionnaire	NA	Physician-diagnosed	Age at diagnosis, race. site, menopausal status, age at first birth, number of births, family history, previous breast biopsy, alcohol, BMI, number of mammograms within the 5-year period prior to one year before diagnosis	6
Soler, 1999, Italy	Case–control	639	<75	Questionnaire	BP ≥ 160/95mmhg	Histologically confirmed	Age, area of residence, education, smoking, alcohol, parity, menopausal status, BMI	7
Talamini, 1997, Italy	Case–control	86	20–74	Questionnaire	NA	Histologically confirmed	Study area, age, education, parity, BMI, menopausal status	6
Moseson, 1993, USA	Case–control	148	22–84	Physician-diagnosed	NA	Biopsy	Age, family history of breast cancer, age at first full-term birth, height, screening variables, null parity, Jewish religion, Latin American birthplace	7
Franceschi, 1990, Italy	Case–control	501	Mean: 45–54	Physician-diagnosed	NA	Histologically confirmed	Terms for medical condition or procedure, age, area of residence, education, age at first birth, menopausal status and, except for severe overweight, BMI	6
Thompson, 1989, USA	Case–control	635	<55	Physician-diagnosed	NA	Histologically confirmed	Age and geographic region.	5

NA, not available; NOS, Newcastle–Ottawa Quality Assessment Scale; BP, blood pressure; DBP, diastolic blood pressure; SBP, systolic blood pressure; BMI, body mass index; USA, united states of America; Mets, metabolic syndrome.

**Table 2:** Subgroup analysis of the relationship between hypertension and risk of breast cancer.

Characteristics	No. of studies	RR (95% CI)	I^2^ (%)	P-value^a^	P-value^b^
Total	30	1.15 (1.08,1.22)	72.30	<0.001	
Geographical region					
America	14	1.18 (1.06,1.31)	72.80	<0.001	0.84
Europe	11	1.16 (1.05,1.29)	78.90	<0.001	
Asia	5	1.07 (0.94,1.22)	29.40	0.23	
Study design					
Retrospective	18	1.29 (1.14,1.47)	77.80	<0.001	0.22
Prospective	12	1.07 (1.01,1.14)	57.30	0.01	
Number of breast cancer cases					
<200	9	1.20 (0.97,1.47)	62.90	0.01	0.98
≥200	20	1.15 (1.07,1.25)	75.40	<0.001	
Data extracted from Mets studies					
Yes	7	1.26 (1.08,1.47)	59.40	0.02	0.38
No	23	1.12 (1.05,1.20)	74.50	<0.001	
Study quality					
NOS < 7	16	1.21 (1.10,1.34)	80.10	<0.001	0.88
NOS ≥ 7	14	1.09 (1.01,1.17)	45.30	0.03	
Definition of hypertension					
≥130/85mmhg	11	1.14 (1.02,1.26)	54.40	0.02	—
≥140/90mmhg	3	2.18 (1.31,3.65)	42.40	0.18	
≥160/95mmhg	3	1.09 (0.91,1.31)	79.70	0.01	
Menopausal status					
Premenopausal	9	0.97 (0.84,1.12)	29.30	0.19	—
Postmenopausal	13	1.20 (1.09,1.31)	63.20	0.001	

RR, relative risk; CI, confidence interval; NOS, Newcastle–Ottawa Quality Assessment Scale; *P*-value^a^, *p* for heterogeneity within each subgroup; *P*-value^b.^, p for heterogeneity between subgroups.
